# A validation study of the Eurostat harmonised European time use study (HETUS) diary using wearable technology

**DOI:** 10.1186/s12889-019-6761-x

**Published:** 2019-06-03

**Authors:** Teresa Harms, Jonathan Gershuny, Aiden Doherty, Emma Thomas, Karen Milton, Charlie Foster

**Affiliations:** 1 Department of Social Science, Centre for Time Use Research, University College London, 55–59 Gordon Square, London, WCH 0NU UK; 2 Nuffield Department of Population Health, University of Oxford, Oxford, UK; 3 Melbourne School of Population and Global Health, University of Melbourne, Melbourne, Australia; 4 Norwich Medical School, University of East Anglia, Norwich, UK; 5 School for Policy Studies, University of Bristol, Bristol, UK

**Keywords:** Time-use diary, Wearable camera, Accelerometer, Physical activity, Data calibration

## Abstract

**Background:**

The central aim was to examine the accuracy of the full range of daily activities recorded in *self-report time-use diaries* against data from two objective passive data collection devices (*wearable camera* and *accelerometer*) serving as criterion reference instruments. This enabled systematic checks and comparisons on the *timing*, *sequence* and *duration* of activities recorded from the three data sources.

**Methods:**

Participants (*n* = 148) were asked to complete a single-day self-report paper time-use diary designed for use in the Harmonised European Time Use Study (HETUS), while simultaneously wearing a camera that continuously recorded images of their activities, and an accelerometer tracking physical movement. In a *reconstruction interview* shortly after the data collection period, participants viewed the camera images to help researchers interpret the image sequences. Of the initial 148 recruits (multi-seed snowball sample, 59% women, aged 18–91, 43% > 40) 131 returned usable diary and camera records (of whom 124 also provided a usable whole-day accelerometer record. We compare time allocation estimates from the diary and camera records, and also match the diary and camera records to the simultaneously recorded accelerometer vector magnitudes.

**Results:**

The data were examined at three analytic levels: *aggregate*, *individual diarist* and *timeslot*. The most important finding is that the estimates of mean daily time devoted to 8 of the 10 main activities differ by < 10% in the camera and diary records. The single case of major divergence (eating) can be explained by a systematic difference between the procedures followed by the self-reporting diarist and the observer coding the camera records. There are more substantial differences at the respondent level, paired t-tests showing significant differences in time spent in the 4/10 categories. 45% of all variation in the accelerometer vector magnitudes in the timeslots is explained by camera and diary records. Detailed activity classifications perform much better than METs as predictors of actigraphy.

**Conclusions:**

The comparison of the diary with the camera and accelerometer records strongly supports using diary methodology for studying the full range of daily activity, particularly at aggregate levels. Accelerometer data could be combined with diary measures to improve estimation of METs equivalents for various types of active and sedentary behaviour.

## Background

### Aims

The CAPTURE-24 project is the first full-scale attempt to test *continuous* diary records against *objective* measures of daily activity recorded in real time. The central aim was to examine the accuracy of activities recorded in *self-report time-use diaries* (TUD) against data from two passive data collection devices (*wearable* cameras and accelerometers) serving as criterion reference instruments. This enabled systematic checks and comparisons on the *timing*, *sequence* and *duration* of activities recorded from the three data sources.

## Literature

Although methodological research into TUD validity and reliability has a long history, most studies have relied on the convergence of multiple non-criterion variables [1–5]. The emergence of wearable sensors presents an opportunity to employ objective criterion measures to test self-report TUDs.

Some public and population health researchers analyse data from time-use surveys (TUS) or use TUDs as a data collection method [6–10]. However, they are not routinely employed to estimate the extent and distribution of time devoted to all physical activity (PA) through the entire day across large representative populations. The standard has been to use various PAQs, particularly the International Physical Activity Questionnaire (IPAQ) or its Short Form (IPAQ-SF), despite known shortcomings such as social desirability bias [11–14] leading to very large overestimations of certain types of activities and physical activity energy expenditure (PAEE) [15].

Studying PA as a complex and multi-dimensional behaviour [16] requires careful instrument design, including a clear definition of variables and a systematic approach to selecting direct (objective) and self-report measures [13, 17–20]. PA is typically measured across four dimensions; *type*, *frequency*, *duration* and *intensity* [13, 16, 21]. The social constructs of *where* and *why* (purpose) people engage in PA are additional dimensions [22, 23]. Accelerometers capture PA frequency, duration and intensity, but not its type or purpose. Self-report TUDs record the frequency, duration, location, type and purpose of PA, although can only estimate PAEE.

### Significance and contribution to the field

Population health studies show a well-established association between decreasing levels of PA and chronic diseases and conditions. This provides a strong public health-based motivation and justification for testing and developing research designs and associated instruments that capture precise measures of daily PAEE, including crucial contextual information such as purpose, type and location. The Multinational Time Use Study (MTUS) [24] includes TUS with detailed information on people’s activities across 24 h periods (including PA), that can be used for historical analysis. However, this requires testing *continuous* TUD records against *objective* measures of daily activity recorded in real time.

## Methods

### Design

The study design and associated standard operating procedures (SOPs) were based on findings of a pilot study (*n* = 14) [25]. A member of the research team met with participants to explain the project purpose, gain written informed consent, complete a short demographic questionnaire (including self-reported height and weight to calculate body mass index (BMI)) and deliver the three instruments and instructions on how to use them. On the allocated data collection day, participants completed the TUD and wore the camera and accelerometer. A few days later, participants met with a researcher for a ‘reconstruction interview’ and received a £20 shopping voucher after its completion.

### Sample and setting

The CAPTURE-24 sample of 148 adults from the UK county of Oxfordshire, returned 124 complete TUD, camera and accelerometer records, and 131 TUD/camera pairs. In order to maximise participant variability, recruitment involved a range of sources (professional networks, free online advertisements, posters, leisure clubs, word of mouth and emails to an authorised list of volunteers). Where possible, researchers made visits in person to promote recruitment. University-educated participants were over-represented (72%) as compared to the UK population (28%). More women than men completed all instruments (62%) and the age distribution was skewed towards the young, with 74 respondents aged 18–39, 34 aged 40–59 and 23 aged 60 and older.

### Instruments

This study used the UK version of the Harmonised European Time Use Study (HETUS) TUD [26]. Participants completed the diary in their own words, starting at 4:00 am, covering 24 h in 10 min intervals (‘timeslots’). The TUD has six *recording fields*: primary and (up to three simultaneous) secondary activities (free text) plus co-presence, location/travel mode, technology use, and enjoyment (pre-coded). The TUD record is a sequence of *episodes*, defined as a period during which none of the six fields change. Using 10 min intervals potentially limits the reporting of short-duration (e.g. visiting the bathroom, checking text messages) or momentary activities (e.g. taking medication, using an ATM), so participants were asked to record these as secondary activities within the appropriate timeslot. Respondents were asked to complete the TUD as frequently as possible during the data collection day. The diary takes round 20 min to complete.

Wearable cameras (e.g. SenseCam) have been used to investigate daily activities and routines [27] and as criterion reference instruments to compare self-report travel diary data [28] and accelerometer counts [29, 30]. Results suggest that wearable cameras are a useful tool for identifying over-reporting of socially disable activities [27] and for better understanding health behaviours in free-living conditions [28–30].

Participants wore the Autographer (formerly SenseCam) on a lanyard or clipped to their clothing during waking hours. The camera captured images (no sound) automatically at 20–30 s intervals (varying according to ambient light and movement) from the participant’s point of view, delivering 1500–2500 images during the wearing period. As the camera is not waterproof, participants were asked not to wear it whist bathing or swimming. Occasionally, clothing or hair obscured the lens, or data were lost when the camera was turned off for various reasons (e.g. for privacy or unintentionally).

The *Axivity AX3 band accelerometer*, released in 2012, is a continuous logging accelerometer designed for various applications including PA monitoring and classification, and motion analysis [31–34]. This particular device was chosen because of its large scale use in the UK Biobank study (> 100,000 respondents). Participants wore the accelerometer for at least 24 h on their dominant hand (wrist). As the AX3 is robust and waterproof, participants were able to wear it continuously. The AX3 is compliant with the OpenMovement data format, has configurable sample rates, adjustable sensitivity and a low power mode. The sample rate of 400 Hz gives a battery life of 5 days and the in-built clock and calendar accurately time-stamp the recorded triaxial acceleration data.

**Fig. 1:**
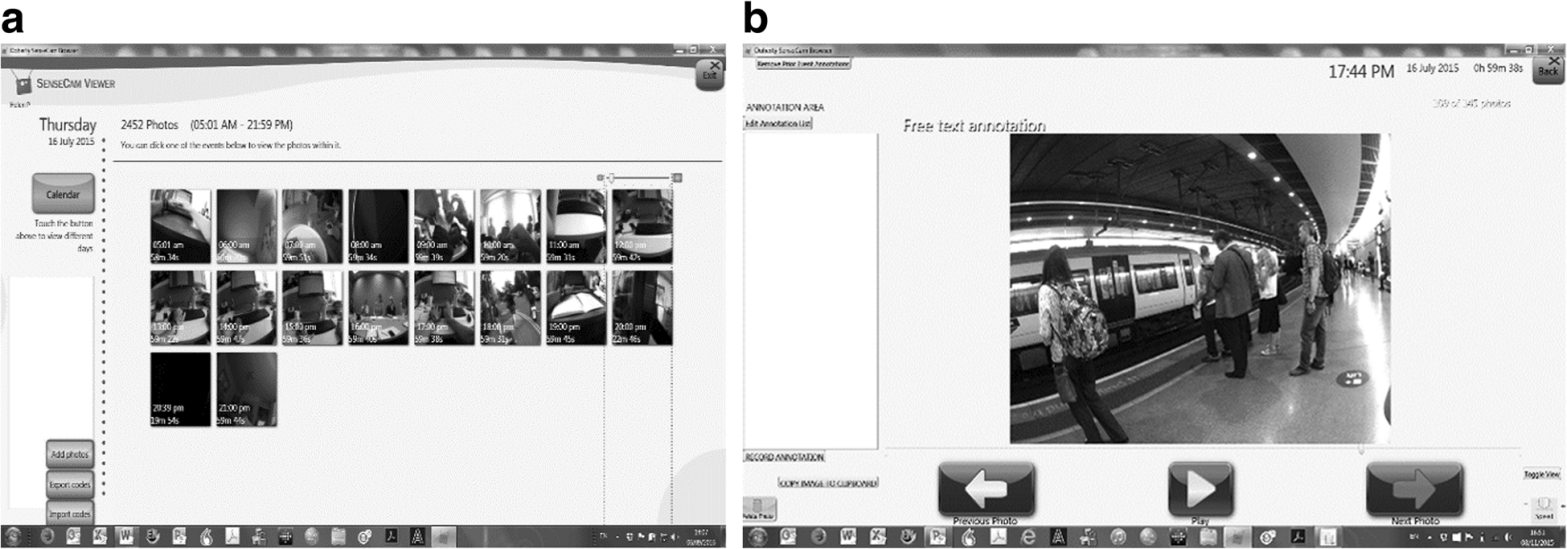
The browser images in thumbnail (**a**, left) and single-image (**b**, right) modes

Shortly after the data collection period (maximum 4 d), participants viewed the camera images in a recorded face-to-face *reconstruction interview* similar to a traditional ‘yesterday’ recall interview, but with higher validity and reliability due to the image prompts [13, 17, 19, 35]. Before the interview, the investigator downloaded the images into a bespoke browser [36] and invited the participant to view and delete (in private) any unwanted images. The interviewer discussed the sequence of images with the participant and kept detailed notes to assist with the data coding process (Fig. 1). Most interviews lasted 50–60 min.

### Ethical considerations

The study received ethical approval from University of Oxford Inter-Divisional Research Ethics Committee (IDREC, reference number SSD/CUREC1A/13–262). The study investigators followed appropriate protocols for conducting research using wearable cameras [37–39].

## Data coding and analysis

Using the data as a test of TUD accuracy made it essential to code the diary and image data independently, so the two coding exercises were carried out separately, approximately 4 months apart. The large number of respondents, combined with the anonymity of the data files, meant that the coder could not connect the TUDs with the corresponding image files, minimising contamination of the image data by the diary records.

### TUD coding

The HETUS activity coding lexicon is hierarchical, the 4-digit level including ~ 250 activities, with 10 single-digit categories for primary and secondary activity fields. The TUD also contains data fields recording co-presence, location or travel mode, technology use, and enjoyment [26]. The coder categorised the diarist’s activities across all six fields, then determined the start and end time of each episode. The final coded diary data file comprised, for each participant, a sequence of episodes of varying lengths, starting at 04.00 with a total duration of 1440 min.

### Camera image coding

The coding procedures used for the TUD were applied as far as possible to the raw camera images (excluding the enjoyment field). Activities were classified as episodes and assigned a HETUS code if they continued for 3 or more images (~ 1 min), whilst activities that lasted just 2 images were grouped with the activity immediately preceding them. The interview notes allowed missing/black images to be coded and for additional field information (e.g. secondary activities, location and others present) to be included.

For the purposes of analysis described below, the initial 1 min timeslots coded in the image files were concatenated to 10 min to correspond with those in the TUD. When multiple activities were recorded within the same 10 min timeslot, the longest was treated as the primary activity and the others coded as secondary.

### Accelerometer data extraction

The accelerometer data processing followed the procedures used by the UK Biobank accelerometer data processing expert group, including device calibration to local gravity, and resampling to 100 Hz [34]. The analyst calculated the sample level Euclidean norm of the acceleration in x/y/z axes and removed machine noise using a fourth order Butterworth low pass filter with a cut-off frequency of 20 Hz. In order to extract the activity-related component of the acceleration signal, one gravitational unit from the vector magnitude was removed, with remaining negative values truncated to zero. Device non-wear time was automatically identified as consecutive stationary episodes lasting at least 60 min.

### Estimating PAEE

Accelerometer measures that represent total activity volume, such as average vector magnitude, are appropriate measures of PAEE [34, 40, 41]. Each signal was summed over 1 min. The sample level data were aggregated into 10 min epochs for summary data analysis, maintaining the average vector magnitude value over the epoch (in milli-gravity units).

### Estimating METs

The final section of the analysis attempts to explain variation in the accelerometer record by differences in the concurrent activities in the camera and diary records. We deploy for this purpose the associations of time use categories with levels of physical activity (METs) reported by Tudor-Locke and colleagues [42, 43] and discussed elsewhere in this issues.

### Analytic methods

The research data were examined at three analytic levels: *aggregate*, *individual diarist* and *timeslot* (10 min interval). At the aggregate and individual levels we focus on the time spent in the 10 single-digit activity groups, comparing the TUD and the camera measures. At the aggregate level, we report means and standard deviation, and calculate percentage difference between the means. At the individual level we consider correlation and t-test results. The timeslot analysis uses OLS to compare METs in each timeslot with the relevant accelerometer vector magnitude, as well as a Boolean extension of OLS to decompose the variation in the accelerometer scores by the detailed time-use categories.

## Analysis

The aggregate analysis reveals how accurately TUDs represent sample or sub-sample durations in particular activities, while the individual (diarist/participant) indicates how well each TUD represents the corresponding camera records. Analysing the 10 min timeslots permits the comparison of different types of activity with the corresponding PAEE derived from the accelerometer data. The main conclusions relate to the aggregate analyses, which are directly applicable to population health studies. The timeslot analysis uses all three instruments to provide evidence of the real-time coincidence between the camera and diary records of similar active and sedentary activities.

### Aggregate analyses

Table 1 includes both aggregate (whole sample) and individual analyses calculated from the *primary activity* fields of the 131 TUDs, with 10 single-digit activity groups summarised from the 149 activity categories actually deployed in the TUD coding, and the 162 in the camera. The left-hand shaded panel shows the aggregate means and standard errors of the two indicators (TUD and camera) while the right-hand panel provides pairwise comparisons.

**Table 1:**
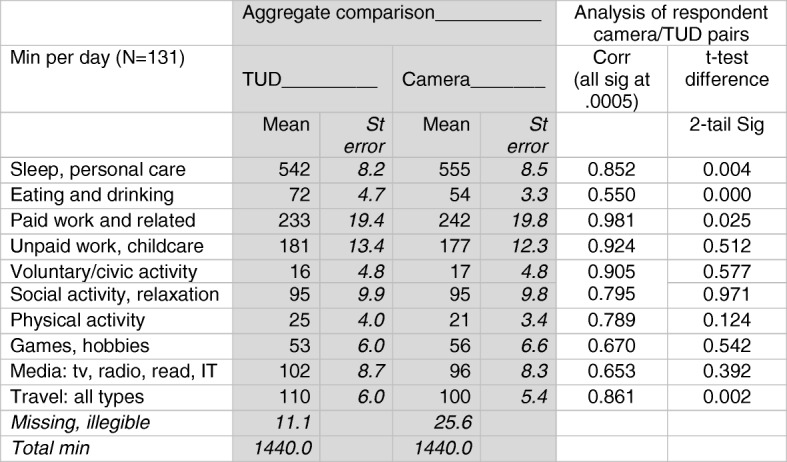
Aggregate- and respondent-level comparisons of camera and TUD activity totals

The aggregate comparison, with two exceptions, shows similar means, differing by <10% of the estimates for *games and hobbies*, *social activity and relaxation*, *media use*, both *paid* and *unpaid work*, *sleep and personal care*, and *travel*. Although *physical activity* exhibits a larger (18%) gap, the strong correlation between the two measures and the statistically non-significant difference between them, renders this unproblematic. The only divergence of concern is in the *eating* category.

This divergence can be explained in terms of differences between the primary/secondary hierarchy in the respondent’s TUD record and the sequence of episodes constructed independently by the camera image coder. The diarist records daily activities as successive events (e.g. *bathing*, then *preparing breakfast* then *eating breakfast*) that are reflected directly in the coding. Secondary or simultaneous activities may occur whilst eating breakfast, such as *chatting with family*, *checking emails* or *reading the paper*. The camera coding protocol instructing that three successive images constitute an episode, may result in the same breakfast activity occurring as multiple eating episodes interspersed with chatting, checking emails and reading the paper.

Table 2 compares the mean duration of 54 min of *eating* as the main activity, as recorded in the camera data, with the aggregate mean total of 10 min timeslots during which eating is mentioned either as a primary or one of the secondary/simultaneous activities in the TUD. The 115 min is obviously an overestimate of the eating duration, since many of the secondary/simultaneous activities are likely to be short duration episodes of snacking or drinking. The gap between the TUD (54 min) and camera (72 min) of eating should be interpreted in terms of the very high occurrence of simultaneous activity associated with eating. The size of the gap between the two eating estimates is much larger than those for watching television and reading.

**Table 2:** Time-reporting hierarchy in the camera records (mean min/d)

Activity	Eating	TV	Reading
Reported as primary activity	54	64	30
Reported as primary or secondary simultaneous activities	115	101	43

**Fig. 2:**
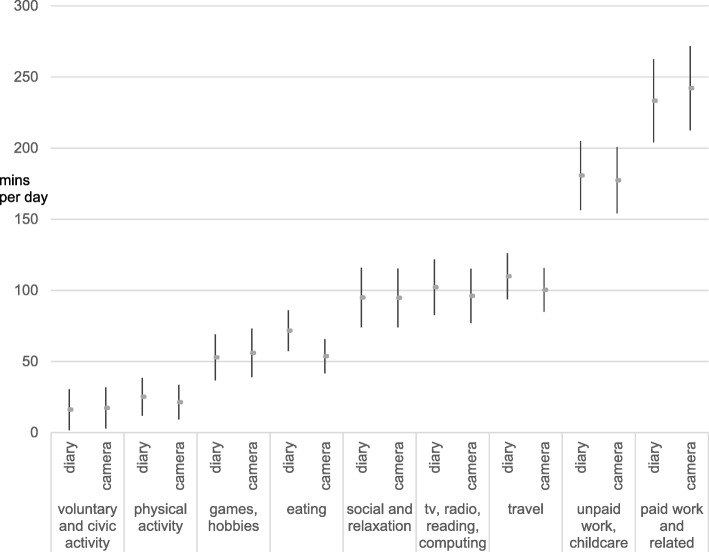
Comparison of TUD and camera estimates: Aggregate mean activity time and 95% confidence intervals (*N* = 131)

Figure 2 compares the camera and TUD means of nine of the 10 activity categories listed in Table 1. Sleep (omitted) has 95% diary/camera confidence intervals of 16.1/16.7 min. The 10% overestimation of time devoted to PA recorded in the TUD compared with the camera may have a similar multiple activity explanation to the eating example in Table 1, although this is a marked improvement over the much larger overestimates (often doubling the participation rate as compared to the diary) associated with questionnaire-based reports of PA [14, 44].

### Accelerometer measures

**Fig. 3:**
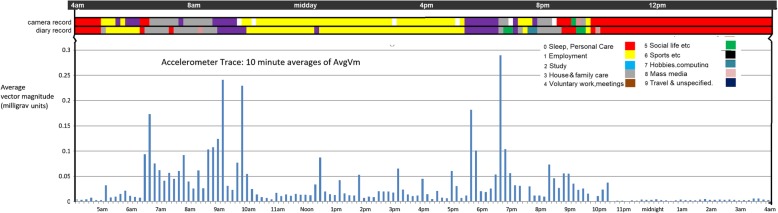
Example camera and diary sequence and accelerometer trace

The accelerometer data (the second criterion measure) have a less direct relationship to TUD estimates of time spent in different activities. This analysis relates the TUD and camera records of the activities in each 10 min timeslot to the accelerometer measure for the same timeslot. Figure 3 shows an example from the pilot sample illustrating the correspondence of the three measures. We see the respondent rising soon after 5 am and doing paid work at home, before waking her children and preparing them for school. Between 9 am and 10 am she is traveling to work (accelerometer spikes for hurrying to and from bus-stop). Through the afternoon at work, we observe occasional spikes of PA representing walking up and down stairs, then we see travel home with a similar pair of walking spikes. Note the close-to-zero actigraphy scores for the night-time sleep period.

The analysis involves two different OLS modelling techniques. The ‘METs-Based Model’ replaces each of the coded activities of the TUD and camera records with its equivalent METs.

The first two substantive columns of Table 3 describe the results from an analysis of a dataset comprising all of the 10 min timeslots for which all (*N* = 17,125) of the three measures (camera, TUD and accelerometer) are non-missing. The left-hand column relates to the METs-based model, regressing each timeslot’s accelerometer total on to the MET score attached to the main activity in that same timeslot. The TUD MET score explains 25% of the total observed variation (adjusted R^2^) in accelerometer scores. The camera MET score explains 27%.

**Table 3:** Comparison of METs-based and detailed activity OLS approaches to explaining variation in accelerometer records

(adjusted R Squared)	METs–based models	Dummy variables models	DVM TUD = Camera
*N*	*17,125*	*17,125*	*11,898*
TUD alone	0.250	0.412	0.531
Camera alone	0.268	0.442	0.525
Camera + TUD	0.298	0.450	0.535

Do the TUD and camera MET scores explain the same parts of the variation in the accelerometer scores? The bottom row of Table 3 provides adjusted R^2^ for a multiple regression of the accelerometer score on to *both* the camera and TUD METs. These together explain 30% of the accelerometer variation. Therefore, 3% of the accelerometer variation is explained by the TUD-based METs but not by the camera-based METs. 5% of the accelerometer variation is explained by camera-based METs but not the TUD METs, while the remaining 22% is explained jointly by both indicators. This suggests that the camera and TUD are mostly explaining the same component of the variation in the accelerometer scores. (Or in terms of Fig. 3: it demonstrates that the accelerometer spikes are in general associated with *both* the camera and the diary event sequence.)

However, 70% of the accelerometer variation is *not* explained by either or both estimators, and 75% is left unexplained by the TUD MET scores on their own. This unexplained variance has four possible components: (a) there is some variation in PAEE *within* the 10 min timeslots; (b) part may be explained by shortcomings in the attribution of MET scores to the 10 min timeslots in each activity category; (c) some may relate to a mis-classification of the main activity during some 10 min timeslots; and (d) some may relate to the placement of the accelerometer on the dominant wrist (hence more movement will be attributed to tennis than to football participation, although both fall into the same HETUS activity category).

The study data can be used to partially decompose this unexplained variation deploying a second OLS technique; binary categorical, ‘Boolean-’ or ‘dummy-variable’ regression. The 150 activity types registered in the TUDs, and the 163 in the camera record are represented as 149 and 162 ‘0/1’ or ‘dummy’ variables (the 150th and 163rd types, respectively, being represented by the ‘default’ case where all the 0/1 dummies are set to 0). The multiple regression coefficients then represent the mean accelerometer counts associated with the PA in various activities.

The TUD registered PA variation now explains 41% of the variation and the camera 44%: jointly they explain 45% of the variation. The difference between the pairs in the first two rows of Table 3 represent that part of the variation in the accelerometer counts explained by the activity categories, but not captured by the METs attributions implemented in this paper. The as yet unexplained 55–59% of the accelerometer variation may reflect differences in the PAEE associated with different 10 min timeslots in particular activities (partly due to the long observation period) or mis-classification of activities.

Given the multiplicity of simultaneous activities associated with each individual, the same activity might potentially be described in different ways by participants. For this analysis, the ‘correct activity classification’ is achieved when the camera and TUD records classify the timeslot similarly. 75% of the 17,125 timeslots are correct at the (10-category) 1-digit level, and 71% at the 2-digit classification level. The third substantive column of Table 3 displays these 11,898 ‘2-digit correct’ timeslots. The TUD evidence now explains 53% of the variation in the accelerometer counts, with the TUD now performing slightly better than the camera. The difference between the second and third columns represents the misclassification component of the unexplained variance, while the remaining unexplained 47% of the accelerometer count variation is due to variation in PA intensity within the timeslots, a result of the granularity of the diary record (i.e. the 10 min timeslot).

## Discussion

This paper makes an important contribution to existing public health literature. The overall purpose of the project was to test the self-report diary method of capturing time-use data against records of activity that are sufficiently objective to be considered as *criterion tests*. Our own comparison of time-use diary-based accounts [25] confirms previous estimates that the PAQ approach roughly doubles the actual level of self-reported PA [5, 15]. Analysis of combined diary and accelerometer data [8] has established that time-use diary records provide measures of sedentary behaviour, as well as light, moderate and vigorous PA that are superior to available alternative methods, but do not specifically link episodes of moderate to vigorous and light physical activity to particular activity categories (e.g. gardening, physical childcare, household work). A recent paper [45] compares travel diaries to both camera and accelerometer evidence. There is no previous paper that comprehensively assesses the convergence of self-report TUD records with camera and accelerometer measures, with each diary event classified across the *full range of daily activities*.

### Limitations

We claim, on the basis of the evidence presented in this paper, that TUDs provide a reasonably accurate and unbiased record of daily activity. However, the relatively poor performance of the METs attributions compared with the potential of the diary and the camera records to explain the variation in the accelerometer record, point to a limitation of the relatively small sample size. A considerably larger sample will be required to improve the calibration of METs attributions to TUD activity records.

Despite the close similarity in aggregate activity totals, there is evidence of quite substantial differences, at the individual level, between the TUD and camera: 25% of the timeslots are coded differently at the 1-digit level in the TUD and camera records. However, the demonstration (Table 3) that the diary and camera mostly explain the same parts of the accelerometer variation is reassuring. Individual level differences are, in effect, self-cancelling at the aggregate level, which is likely due to random recall errors in episode start and finish times. The lower levels of explanation of accelerometer data from the METs compared with the specific activity classifications suggests the current attribution of METs to diary activities has room for improvement.

Nevertheless, the strong similarity between the camera and diary results suggest that a larger follow-up could be undertaken with diaries and accelerometers alone, without the (costly) camera records.

## Conclusions

We demonstrate that self-report time-use diaries provide a reliable basis for the accurate estimation of time-use patterns, without evidence of bias by educational level. By direct inference, we conclude that when collected from representative samples, time-use diaries can validly and reasonably reliably represent the time-use of large populations. This is an important advance on the previous time-diary evaluation literature, insofar as it relies not on a priori reasoning but on comparisons with unimpeachable criterion data.

Understanding how large representative populations spend their time allows public health researchers to examine PA across the full 24 h covered by the TUD. This includes the occupational, domestic, transport and leisure time physical activity domains, as well as sleep quality and duration and sedentary time. Comparing the diary with the camera and accelerometer records strongly supports using diary methodology for studying the full range of daily activity, particularly at aggregate levels. Accelerometer data could be combined with diary records to improve the estimation of METs equivalents for various types of active and sedentary behaviour.

## Abbreviations

BMI: Body mass index
CAPTURE-24: Comparing Annotated Pictures with Time-Use Diaries’ Recording of Events over 24-h
HETUS: Harmonised European Time Use Study
IPAQ: International Physical Activity Questionnaire
IPAQ-SF: International Physical Activity Questionnaire Short Form
PA: Physical activity
PAEE: Physical activity energy expenditure
PAQ: Physical Activity Questionnaire
SOP: Standard operating procedure
TUD: Time-use diary
